# Protein Interactions and Regulation of EscA in Enterohemorrhagic *E. coli*

**DOI:** 10.1371/journal.pone.0085354

**Published:** 2014-01-13

**Authors:** Ching-Nan Lin, Wei-Sheng W. Sun, Hui-Yin Lu, Swee-Chuan Ng, Ying-Shu Liao, Wan-Jr Syu

**Affiliations:** 1 Institute of Microbiology and Immunology, National Yang-Ming University, Taipei, Taiwan, Republic of China; 2 Institute of Biochemistry and Molecular Biology, National Yang-Ming University, Taipei, Taiwan, Republic of China; 3 Taiwan International Graduate Program in Molecular Medicine, Academia Sinica, Taipei, Taiwan, Republic of China; Centre National de la Recherche Scientifique, Aix-Marseille Université, France

## Abstract

Infections caused by enterohemorrhagic Escherichia coli (EHEC) can lead to diarrhea with abdominal cramps and sometimes are complicated by severe hemolytic uremic syndrome. EHEC secretes effector proteins into host cells through a type III secretion system that is composed of proteins encoded by a chromosomal island, locus for the enterocyte effacement (LEE). EspA is the major component of the filamentous structure connecting the bacteria and the host's cells. Synthesis and secretion of EspA must be carefully controlled since the protein is prone to polymerize. CesAB, CesA2, and EscL have been identified as being able to interact with EspA. Furthermore, the intracellular level of EspA declines when cesAB, cesA2, and escL are individually deleted. Here, we report a LEE gene named l0033, which also affects the intracellular level of EspA. We renamed l0033 as escA since its counterpart in enteropathogenic E. coli has been recently described. Similar to CesAB, EscL, and CesA2, EscA interacts with EspA and enhances the protein stability of EspA. However, EscA is also able to interact with inner membrane-associated EscL, CesA2, and EscN, but not with cytoplasmic CesAB. In terms of gene organizations, escA locates in LEE3. Expression of EscA is faithfully regulated via Mpc, the first gene product of LEE3. Since Mpc is tightly regulated to low level, we suggest that EscA is highly synchronized and critical to the process of escorting EspA to its final destination.

## Introduction

Enterohemorrhagic *Escherichia coli* (EHEC) O157:H7 [1], [2] forms typical histological lesions termed attaching and effacing lesions on the large intestine tissue of infected individuals. This lesion-generating capability is attributed largely to a set of virulence genes clustering on the bacterial chromosome that is called the locus of enterocyte effacement (LEE) [3], [4], [5], [6]. The LEE contains 41 open reading frames (Orfs) that are largely organized into five poly-cistronic operons. These Orfs have been studied in detail to a variety of different degrees [7] and the better characterized gene products have been categorized into a number of groups, namely components of a type III secretion system (TTSS) (Sep and Esc), secreted translocators and effector proteins (Esp), chaperones (Ces), regulatory factors (Ler, GrlA, GrlR, and Mpc), an adhesin (Eae) and a translocated receptor (Tir). Gene *l0033* in EHEC is among the least well characterized genes and the equivalent genes in *Citrobacter rodentium* and enteropathogenic *E. coli* (EPEC) have been shown to play critical roles in the TTSS [8], [9]. However, information on the mechanism of action of this gene still remains limited.

TTSSs exist in several Gram-negative bacteria that are either pathogenic or symbiotic [10]. The main function of any TTSS is to efficiently and specifically deliver effector proteins from the bacterial cytoplasm into the eukaryote target cell cytoplasm by a direct contact through a syringe-like conduit. In the case of EHEC, one phenotype after delivery of the effector proteins is an induction of rearrangement of the cellular actin by altering the signaling pathways of the infected cells [11]. These alterations result in the formation of pedestal structures. The syringe-like conduit formed is evolutionarily similar to that associated with flagella systems [12]. Ultra-structurally, the whole structure is organized into a needle complex and a basal body. The needle complex of EHEC consists of EspA in the filamentous portion with the tip being EspA in complex with EspB and EspD. On the other hand, the basal body structure consists of an inner membrane ring and an outer membrane ring [13]. In addition to the structural consideration described above, in order to assemble a mature and effective secretion apparatus, timing is highly critical. It is believed that the needle complex is secreted by the TTSS and is then assembled onto the basal body to produce the distal elements of the apparatus [14].

To better understand the mechanistic activity of the TTSS in EHEC, it is highly important to characterize the functions of the less well understood genes in these operons because no gene within the LEE island is dispensable if the TTSS is to be functional and effective [8]. *Orf l0033* of EHEC consists of 378 base-pairs and this encodes a protein of 125 amino acids with a calculated molecular weight of 14.66 kDa. By protein sequence comparison, L0033 shares 100% identity with a counterpart in EPEC and 95% identity with that of a counterpart in *C. rodentium* [9]. Furthermore, there are no other homologues found outside the LEE family. Recent studies in EPEC [9] have confirmed previous findings for *C. rodentium* [8] whereby the counterparts of *l0033* are essential for the secretion of both translocator and effector. Since the gene in these two strains has been renamed *escA*, we will from now on refer to *l0033* as *escA* to allow easier discussion. Moreover, no functional and evolutionary origin for EscA has been suggested based on previous bioinformatics analysis of the LEE [15]. Using yeast two-hybrid system analysis [16], no LEE protein has been found to interact with EscA. However, in contrast to the yeast two-hybrid results, in a recent bacterial pull-down assay EscA had been found to interact with EscC [9]. We have therefore reasoned that there must be additional function(s) carried out by EscA. In this study, our findings suggest that EscA is able to affect the stability of the EspA. In addition to this, EscA was found to interact independently with two EspA-binding proteins (CesA2 and EscL) and EscN, but not a third EspA-binding protein (CesAB). Finally, when regulation in EHEC is examined, it is interesting to note that the expression of EscA has been linked to the presence of Mpc, a protein that is absolutely needed for TTTS functionality; however, this protein is tightly regulated and maintained a low level.

## Materials and Methods

### Bacterial strains and culture conditions

EHEC O157:H7 (ATCC strain 43888) [17] was used as the parental strain for creating specific gene deletions and is referred to here as the wild-type (WT) strain. *E. coli* K-12 strain JM109 (New England Biolabs) was routinely used for DNA cloning and manipulation. Bacteria were regularly grown at 37°C aerobically in Luria-Bertani (LB) broth (Difco). Media were supplemented with antibiotics when necessary: ampicillin, 100 µg/ml; chloramphenicol, 25 µg/ml; and tetracycline, 10 µg/ml. To stimulate EHEC to express the LEE proteins, M9 minimal medium (Difco) in the presence of 5% CO_2_ was used for bacterial cultivation [18], [19]. When grown in LB under standard conditions, LEE expression is repressed [19].

### Primers and expression plasmids

All primers used in this study are listed in Table 1. Plasmids encoding EspA and EspB were constructed by inserting PCR products of the entire Orf (without the stop codon) into pQE60 (Qiagen). The resulting plasmids (pQE-EspA and pQE-EspB) encode the target proteins fused with a hexahistidine (His_×6_)-tag at the C-terminus [20]. To express EscA, EHEC chromosomal DNA was amplified by PCR using the paired primers PEscAF and PEscAR. The PCR product was digested with *Bam*HI and *Bgl*II, and subsequently ligated into the same enzyme-restricted pQE60 to give pQE-EscA. The plasmids pQE-Mpc, pQE-EspF, and pQE-Map were constructed similarly except for using different primer pairs and a second set of restriction enzymes (*Nco*I/*Bgl*II).

**Table 1:** Primers used and their sequences.

Name	Sequence (5′ to 3′)	Used for
EscAF	GAGGATCCATGTTGGACAGAATTTTATC	pQE-EscA
PEscAR	AGATCTGTCAAAGTAATGTTCCTTTATG	
PMpc-F	CCATGGGAATGAATCTTTTAGTTAAAAG	pQE-Mpc
PMpc-R	AGATCTTGATGTCATCCTGCGAACG	
PEspF-F	GGATCCATGCTTAATGGAATTAGTAACGC	pQE-EspF
PEspF-R	AGATCTCCCTTTCTTCGTTGCTC	
PMap-F	CCATGGGAATGTTTAGTCCAATGACAATGGC	pQE-Map
PMap-R	AGATCTCAATCGGGTATCCTGTACATGC	
PescNF	ATGATTTCAGAGCATGATTC	pTZ31-34
PsepQR	ATTCCTGATTAATCACATAC	
PEscA-R-KO	CATACTCAGGCAACCACTTTG	pTZ-33KO
PEscA-F-KO	CATTACTTTGACTAGAGTTTC	
PI-SecI	ATTACCCTGTTATCCCTACAGGCCTCTGCAGTCGAC	
PRBS-HindIII-F	AAGCTTTGAGCGGATAACAATTTCAC	p*T5*-EscA_His-Mpc
PEscA-HindIII-R	AAGCTTCTAGTCAAAGTAATGTTCCTTT	
*escA* RT-F	AGCAGAGCGAACCGATTGAGAGAATC	qPCR
*escA* RT-R	TGTGAATCTAGCAATGAACGCTTTTCC	
*ompC* RT-F	GACGGCCTGCACTATTTCTCTG	
*ompC* RT-R	CTGCGAATGCCACACGGGTC	

To couple the expression of tag-free EspA with His_×6_-tagged EscA, pQE-EscA was used as a backbone, to which a DNA fragment spanning from *T5* promoter to the end of *espA* (without the His_×6_-tag-coding codons) and amplified from pQE-EspA was inserted. The resulted p*T5*-EspA_His-EscA has individual *T5* promoters in front of tag-free EspA and His_×6_-EscA, respectively. A similar strategy was used to construct p*T5*-EspB_His-EscA. The resulted plasmids were screened and clones containing the expected restriction patterns were finally confirmed by DNA sequencing. p*T5*-EscA_His-Mpc was generated by a similar strategy except that the plasmid backbone was pEQ-Mpc and the template used for PCR amplification of the tag-free EscA-coding fragment was pQE-EscA; during the cloning, primers paired for PCR were PRBS-HindIII-F and PEscA-HindIII-R and the restriction enzyme used for digestion and cloning was *Hind*III.

### Constructions of deletion mutant

The method of Gene Gorging was carried out as described previously [21]. This takes advantage of a system that uses the lambda Red recombinase and *in vivo* linearization of a donor plasmid carrying the desired mutation. In brief, a DNA fragment spanning from *sepQ* to *escN* was amplified from the EHEC chromosomal DNA by PCR using the primers of PescNF and PsepQR. The PCR product was subsequently cloned into pTZ57R/T (Fermentas) to result in pTZ31-34. Reverse PCR was then performed using pTZ31-34 as the template and PEscA-R-KO and PEscA-F-KO as paired primers. The PCR product was then self-ligated to produce the *escA*-deleted plasmid. After sequence confirmation, the resulting plasmid was used for PCR re-amplification with primers PescNF and PI-SecI. The PCR product was then cloned into another pTZ57R/T to give pTZ-33KO that was used as the donor plasmid for the subsequent *escA* deletion transformation.

### Expression of recombinant proteins and Western blotting

Anti-EscA antibodies were generated by immunizing mice with Ni^2+^-NTA purified His_×6_-tagged EscA. Commercial rabbit anti-His_×6_ antibodies (Bethyl) were used for detecting His_×6_-tagged proteins. Anti-EspA, anti-EspB, anti-Tir, and anti-OmpC antibodies have been previously described [20], [22].

Sample preparation (from Bacterial lysate and concentrated Supernatant) and Western blotting analysis were carried out as previously described [18]. The final step of blotted membrane development was carried out using Western Lightning™ Chemiluminescence Reagent Plus (PerkinElmer) and after this the signals were captured by exposing the membranes to X-ray films (Fuji).

### EspA and EscA stability assay

The stability assay was carried out as previously described [23]. In brief, bacteria harboring pQE-EspA or pQE-EscA were grown at 37°C aerobically in LB broth overnight. The cultures were then 1∶50 inoculated into M9 medium and incubated at 37°C in the presence of 5% CO_2_ for 5.5 h. Then, IPTG was added to 1 mM and the cultures were further incubated for 0.5 hr. Bacterial protein synthesis was then stopped by adding chloramphenicol to a final concentration at 200 µg/ml. At different incubation times, the cells were sampled, spun down, disrupted by boiling in SDS-containing sample buffer and analyzed for the presence of EspA or EscA by Western blotting using anti-His_×6_ antibodies.

### Affinity chromatography

Affinity binding of His_×6_-tagged proteins to Ni^2+^-NTA agarose beads (Bio-Rad) was carried out as previously described [20] except that the buffer used for equilibration and lysis was 100 mM Tris-HCl, pH 7.4, containing 1 mM PMSF. Wash buffer was consisted of 100 mM Tris-HCl, pH 7.4, containing 50 mM imidazole whereas elution buffer was identical to the wash buffer except that imidazole was added up to a concentration of 250 mM.

### RNA isolation and real-time quantitative RT-PCR (qRT-PCR)

RNA isolation method was carried out as previously described [23]. Briefly, total RNAs were extracted from 6 h-cultured EHEC in M9 by using TRIzol reagent (Invitrogen). After extraction, the RNAs were treated with DNase I (Macherey-Nagel) to remove contaminated DNA and reversed to cDNA with the RevertAid™ First Strand cDNA sysnthesis Kit (Fermentas). Quantitative PCR reaction was carried out in triplicates with the Power SYBR Green Master Mix (Protech) and performed by the Stratagene Mx3000P™ Real-Time PCR System. The primers used for qPCR are listed in the Table 1. Comparative CT was used to determine the relative *escA* mRNA expression fold when normalized to an expression level of internal control gene (*ompC*).

### Bacterial two-hybrid analysis

The system (Stratagene) used has been described previously [18]. In brief, the bait protein generated from pBT plasmid was fused to the C-terminus of aλλcI the target protein, produced from the pTRG plasmid, was fused with an N-terminal RNA polymerase α subunit. The degree of interaction between the bait and target proteins in the system was reflected by measuring the relative β-galactosidase activity [18].

## Results

### EscA of EHEC is essential for TTSS

**Figure 1:**
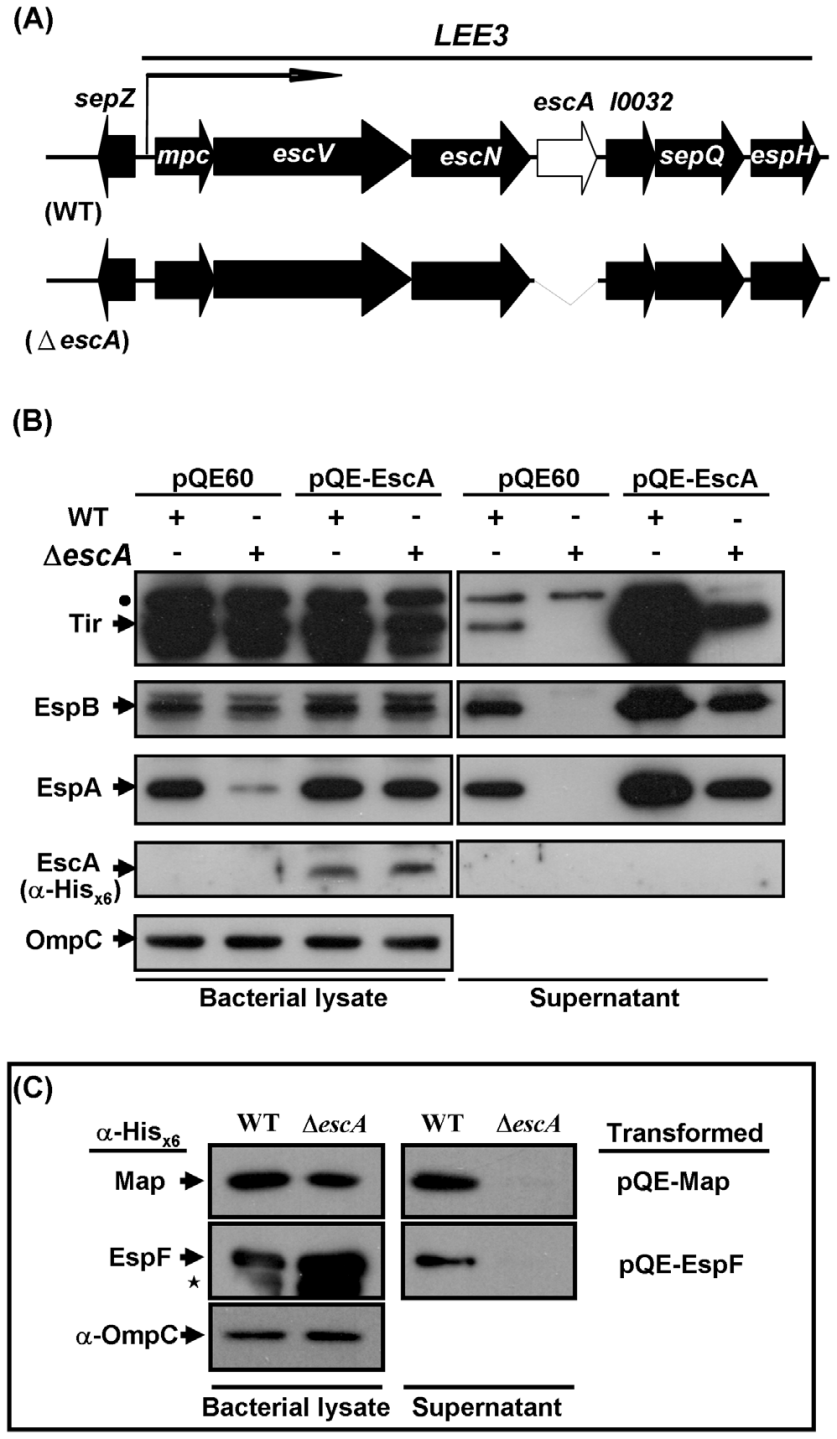
Characterizing the effects of the *escA* deletion on the detection of representative *LEE* proteins. (A) Schematic diagram of the gene organization around *LEE3* and an illustration of how the deletion of *escA* in mutant strain (Δ*escA*) was created. Bent arrow indicates the transcription direction of the *LEE3* operon. (B) Detection of representative LEE proteins (Tir, EspB, and EspA) by Western blotting using specific antibodies. Bacteria transformed with pQE-EscA or control vector (pQE-60) were grown in M9 in the presence of 5% CO_2_. (C) Secretion of effectors Map and EspF also affected by the *escA* delection. Bacteria were separately transformed with pQE-Map and pQE-EspF, and the His_×6_-tagged proteins in the bacterial lysates and the culture supernatants were detected similar to that in (B) except for the excusive usage of anti-His_×6_ antibodies. Bacterial lysate and concentrated supernatant were prepared separately and analyzed accordingly. OmpC was used as the protein loading control for the bacterial lysate samples. Note: dot denotes a detected non-specific signal above the band of Tir whereas asterisk indicates a band presumably arising from His_×6_-tagged EspF that was abundantly expressed.

The *LEE3* operon encodes seven proteins and, among them, EscV, EscN, and SepQ are located on bacterial membrane and are essential for EHEC to deliver proteins from the bacterium into an infected cell [15], [24], [25]. *EscA* is the fourth gene of the *LEE3* operon. To investigate the role of EscA, *escA* was deleted [21] from the chromosome of EHEC to give a deletion strain called Δ*escA* (Figure 1A) hereafter. We examined the expression of a number of representative LEE proteins in this mutant strain. Figure 1B (right panel) shows that EspA, EspB, Tir were absent in the supernatant of the Δ*escA* mutant, a result that is completely consistent with the previous observations in *C. rodentium* [8]. The defective secretion could be restored by complementation with expressed His_×6_-tagged EscA from the plasmid pQE-EscA. When intracellular proteins were examined (Figure 1B, left panel), the EspA level of the Δ*escA* mutant was decreased as compared to that of the WT strain. However, the levels of EspB and Tir were not affected. This decreased level of intracellular EspA could be restored by ectopic expression of EscA when the Δ*escA* mutant was transformed with pQE-EscA. However, this was not the case when transformation was carried out using the vector control, pQE60.

To test whether the *escA* deletion also affects the secretion of effectors Map and EspF, a different strategy of using anti-His_×6_ to detect the plasmid-encoded proteins in the bacterial samples was used. Figure 1C (left panel) shows that both Map and EspF, which were tagged with His_×6_, were well expressed and detected similarly in both bacterial lysates of the parental strain (WT) and the Δ*escA* mutant. However, these tagged Map and EspF were absent in the culture supernatants of the Δ*escA* strain but present unequivocally in that of the WT strain (Fig. 1C, right panel). Therefore, deletion of *escA* disturbs the type-III secretion severely and affects many secreted proteins.

### Decreased stability of EspA in the absence of *escA*

**Figure 2:**
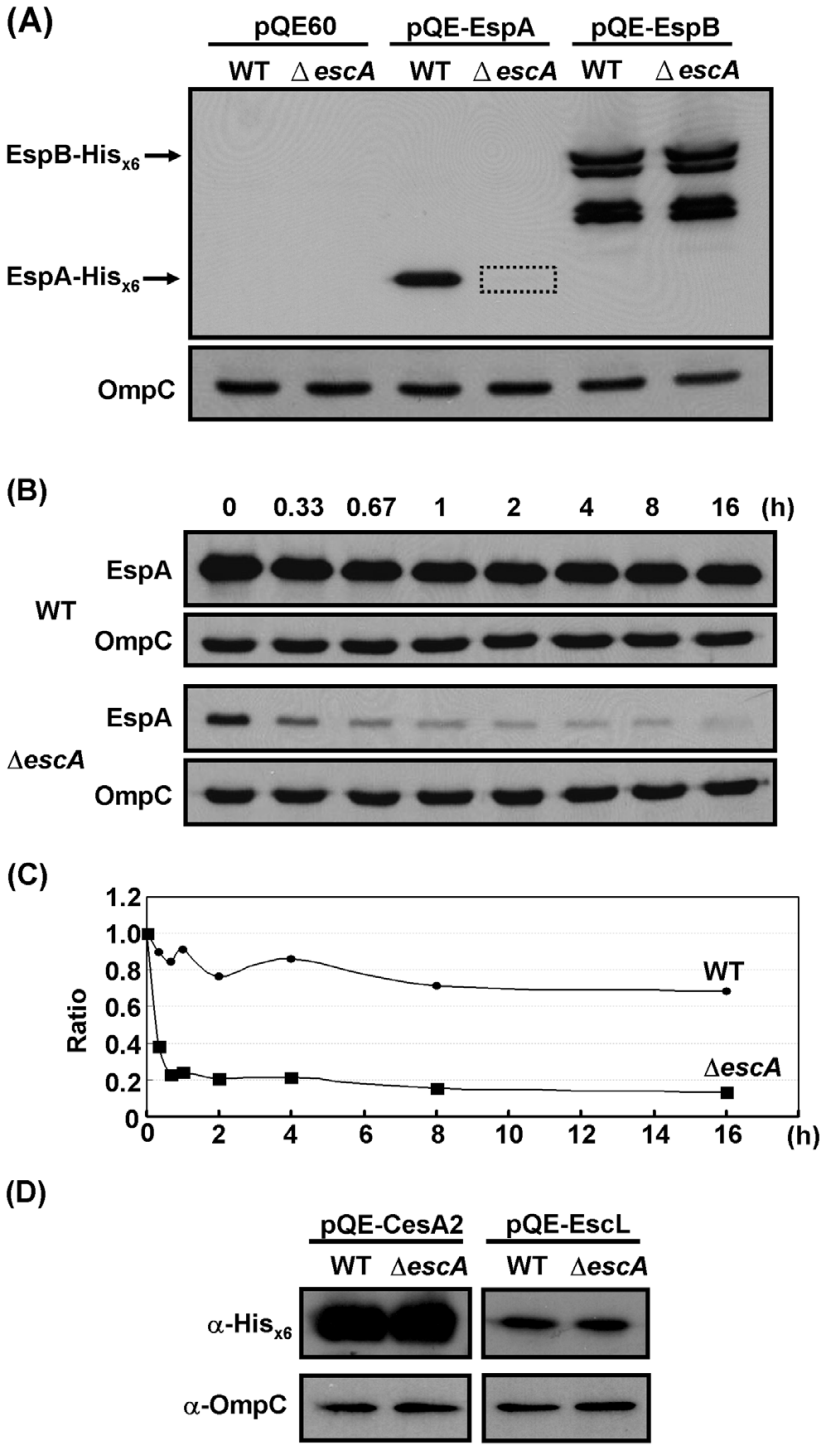
Expression and stability of EspA are decreased in the absence of *escA.* (A) Detection of EspA and EspB that were ectopically expressed and detected by Western blotting using anti-His_×6_. EHEC (WT) and mutant Δ*escA* were transformed with the plasmids indicated and the bacteria were cultured for 6 h at 37°C in M9-5% CO_2_. Proteins from the total bacterial lysates were analyzed by Western blotting using rabbit anti-His_×6_ antibodies. The dotted box indicates the expected banding area of EspA. Multiple bands of EspB detected could be the results of degradation presumably arising from overexpression. (B) Stability of EspA in the bacteria, as reflected by sample analyses over 16 h after *de novo* protein syntheses of bacteria were blocked by addition of chloramphenicol. EspA was analyzed in a way similar to that of (A). (C) The amount of EspA over the time period in (B) was converted into a stability curve by quantifying the band intensities using a densitometer. The EspA intensity at time zero is referred to as 100% while simultaneously detected OmpC was used for loading calibration. (D) Expression of CesA2 and EscL not affected by the lack of EscA in bacteria. Bacteria were transformed with pQE-CesA2 and pQE-EscL, respectively. Bacterial cultures and detection of proteins were carried out similar to that in (A).

To examine the possibility that the decreased level of intracellular EspA is controlled transcriptionally at the promoter, we introduced *T5* promoter-driven expression of EspA-His_×6_ into the Δ*escA* mutant and the parental strain (WT) separately. In these systems, the transcription of the tagged EspA was straightforwardly governed by the *T5 cis*-element of the plasmid. As a control in parallel, EspB-His_×6_ was similarly introduced. After 6 h-cultivation of the bacteria in M9 medium, the expression levels of EspA-His_×6_ and EspB-His_×6_ were determined by Western blotting. Figure 2A shows that the expression levels of EspB-His_×6_ were similar in both the WT and Δ*escA* strains. However, EspA-His_×6_ could hardly be detected in the Δ*escA* strain but was abundantly found in the parental WT strain. These results strongly suggest that the intracellular level of EspA is down-regulated in a novel manner in the Δ*escA* mutant and that transcriptional regulation is unable to account for the differences in expression levels. To confirm that the expression of EspA in strain Δ*escA* is not regulated at the mRNA level, a transcriptional fusion reporter [23] was used. Consistent with the above results, it was found that the β-galactosidase activity derived from the *espA-lacZ* fused transcripts of the WT and Δ*escA* transformants showed no significant difference (data not shown).

To account for the decreased intracellular EspA level in the Δ*escA* strain, we next hypothesized that, in the absence of EscA, EspA might be unstable. To validate this notion, the stability of EspA in the parental WT and that in the Δ*escA* strain were compared after *de novo* protein synthesis in the bacteria was suppressed by treatment with chloramphenicol. Figure 2B shows the Western blotting results for EspA in the bacterial lysates that were harvested at various time points. Apparently, EspA is rather unstable in the Δ*escA* mutant when compared to the parental WT strain. The protein level of EspA in the Δ*escA* mutant was lower than that in the parental strain at time zero and the protein had decayed greatly after 1 h, as revealed by quantitative measurement of the relative protein abundance using densitometry (Figure 2C). In contrast, EspA in the WT strain was maintained at a relatively high level during the sampling period over 16 h.

Whether the unstable EspA in the Δ*escA* mutant could result from a decrease of CesA2 and EscL, proteins known to interact with EspA [23], [26] and EscA (see below), was further examined. By an approach similar to that used in Fig. 2A, we examined CesA2 and EscL that were encoded by pQE-CesA2 and pQE-EscL, respectively, and expressed in the plasmid-transformed Δ*escA* mutant. The results were then side-by-side compared with that seen in the parental WT strain. Figure 2D shows that CesA2 (left panel) and EscL (right panel) were detected indistinguishably in both strains, a result suggesting that CesA2 and EscL are as stable as in the WT strain when the bacteria lack EscA.

### Interaction of EscA with EspA

**Figure 3:**
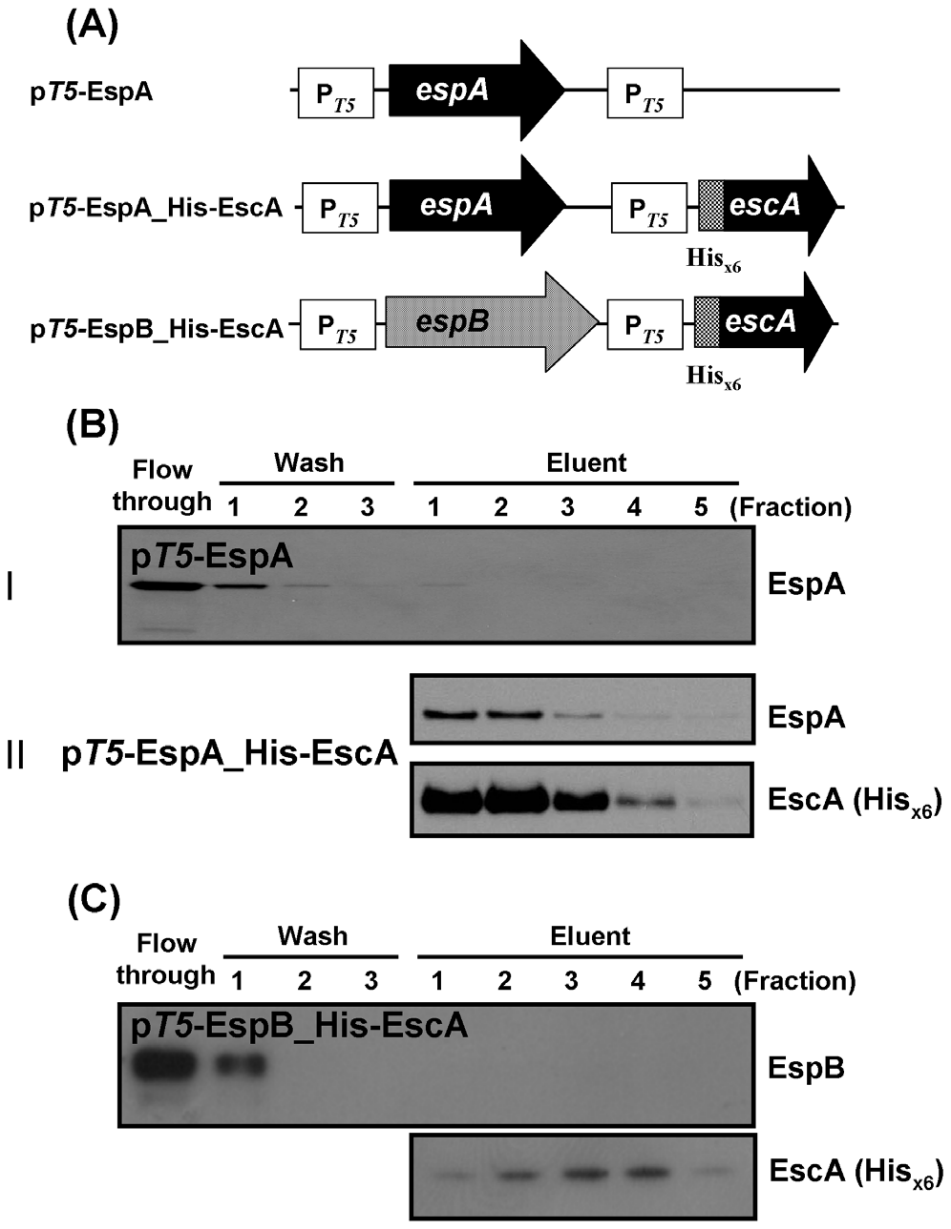
Co-elution of EspA and EscA. (A) Schematic illustration of the expression constructs. (B) Affinity co-elution analysis of Ni^2+^-NTA column-retained proteins. In panels I and II, bacterial lysates from plasmid-transformed *E. coli* JM109 were loaded onto the column. After washing with buffer containing 50 mM imidazole, the retained proteins were eluted with the same buffer containing 250 mM imidazole. The presence of EspA from the individual fractions of the panels was detected by Western blotting using rabbit anti-EspA antibodies. (C) Analysis of EspB in elution fractions of the Ni^2+^-NTA column in the presence of His_×6_-tagged EscA binding. Experiments were carried out in a manner similar to that in (B) except that the bacteria were transformed with p*T5*-EspB_His-EscA and EspB was detected with rabbit anti-EspB antibodies.

To address whether the instability of EspA in the Δ*escA* mutant is due to a lack of protein interaction contributed by EscA, we generated p*T5*-EspA_His-EscA (Figure 3A) to express tag-free EspA and His_×6_-tagged EscA simultaneously. We examined whether the two proteins could be co-purified because of a possible interaction. After washing and affinity purification of His_×6_-tagged EscA from the Ni^2+^-NTA column, EspA was readily detected in the fractions where EscA was eluted (Figure 3B). For a comparison, a similar construct of p*T5*-EspB_His-EscA (Figure 3A) was used to express tag-free EspB and His_×6_-tagged EscA simultaneously. Figure 3C shows that although EspB was found unequivocally in the flow-through fraction of the Ni^2+^-NTA column, it was not detected in the fractions containing the eluted His_×6_-tagged EscA. Therefore, these findings confirm that EscA interacts specifically with EspA.

### Interactions of EscA with CesA2, EscL, and EscN

**Figure 4:**
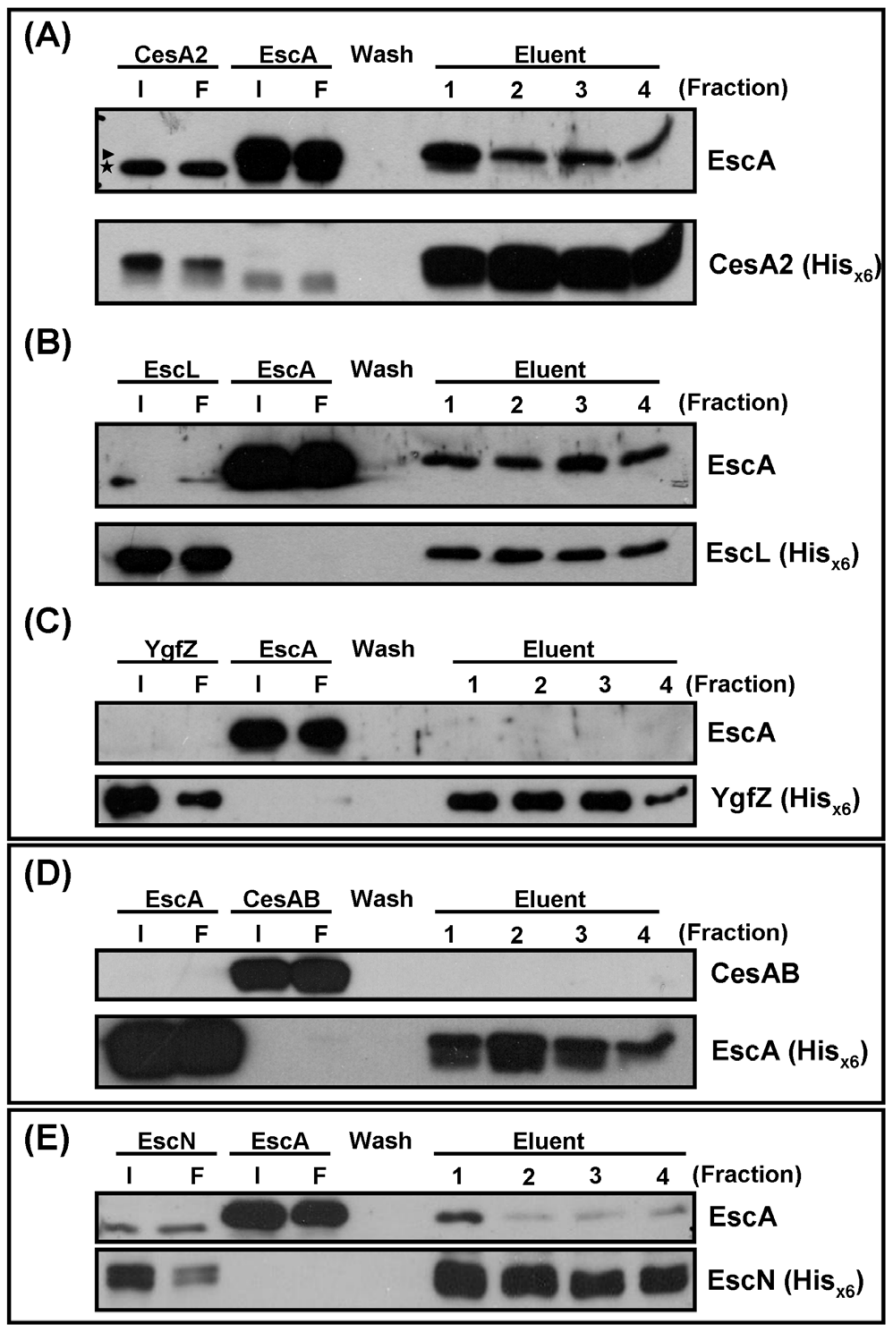
Affinity interaction between EscA with CesA2, between EscA and EscL, and between EscA and EscN as demonstrated by co-elution from a Ni^2+^-NTA column. Experiments were carried out in a manner similar to that in Figure 3 with a slight modification. In essence, a His_×6_ tag was placed at the C-terminal end of CesA2 (A), EscL (B), YgfZ (C), EscA (D), and EscN (E). Bacterial lysate containing the His_×6_-tagged protein was individually applied to the Ni^2+^-NTA column. Then bacterial lysate containing tag-free EscA (A-C and E) or CesAB (D) was applied to the column. After washing and eluting, fractions were analyzed by Western blotting with specific antibodies except that all His_×6_-tagged proteins were all detected by anti-His_×6_. I: input; F: flow through.

The above results indicate that, in the absence of EscA, the stability of EspA was decreased in EHEC and that EscA possessed the abilities to interact with EspA biochemically. These two properties of EscA are similar to those of three other proteins, namely the two chaperons reported for EspA, CesAB and CesA2 [26], [27] and the EspA-binding protein EscL [23]. Thus, we proposed that EscA might conjoin with these proteins in assisting the movement of EspA and perhaps a close interaction between these proteins might exist. To test this notion, pull-down assays were performed to examine a possible interaction between EscA and any one of these three previously known EspA-binding proteins. First, tag-free EscA and His_×6_-tagged CesA2-expressing JM109 were separately cultured and total proteins were subsequently harvested. After binding His_×6_-tagged CesA2 onto Ni^2+^-NTA column and followed by washing, tag-free EscA was subsequently applied to the column. After additional washing and elution, EscA was found to appear with His_×6_-tagged CesA2 (Figure 4A) in the eluent fractions. A similar experiment was carried out to investigate the interaction between EscA and EscL, and Figure 4B shows a positive result that is similar to that of EscA and CesA2. An irrelevant His_×6_-tagged protein (YgfZ) was used as negative control to test for co-elution with EscA using the Ni^2+^-NTA column. Using this control protein, no EscA was detected in the eluent fractions (Figure 4C). On the other hand, a similar attempt to pinpoint whether there is an interaction between EscA and CesAB using the same strategy gave a clearly negative result, which is shown in Figure 4D.

It has been previously reported that CesAB could not interact with EscN. However, the interaction occurs after CesAB interacts with EspA [28]. Therefore, whether EscA could form complex with EscN prior to the interaction of EscA with EspA is then addressed. First, amino acid sequences of CesAB and that of EscA were compared and no primary sequence similarity could be concluded. Second, an interaction possibility between EscA and EscN was tested by using affinity Ni^2+^-NTA column to retain His_×6_-tagged EscN and followed by applying untagged EscA; whether EscA could be co-eluted with EscN was then examined. Figure 4E shows positively that EscA alone could interact with EscN as evidenced by the results of co-elution.

**Figure 5:**
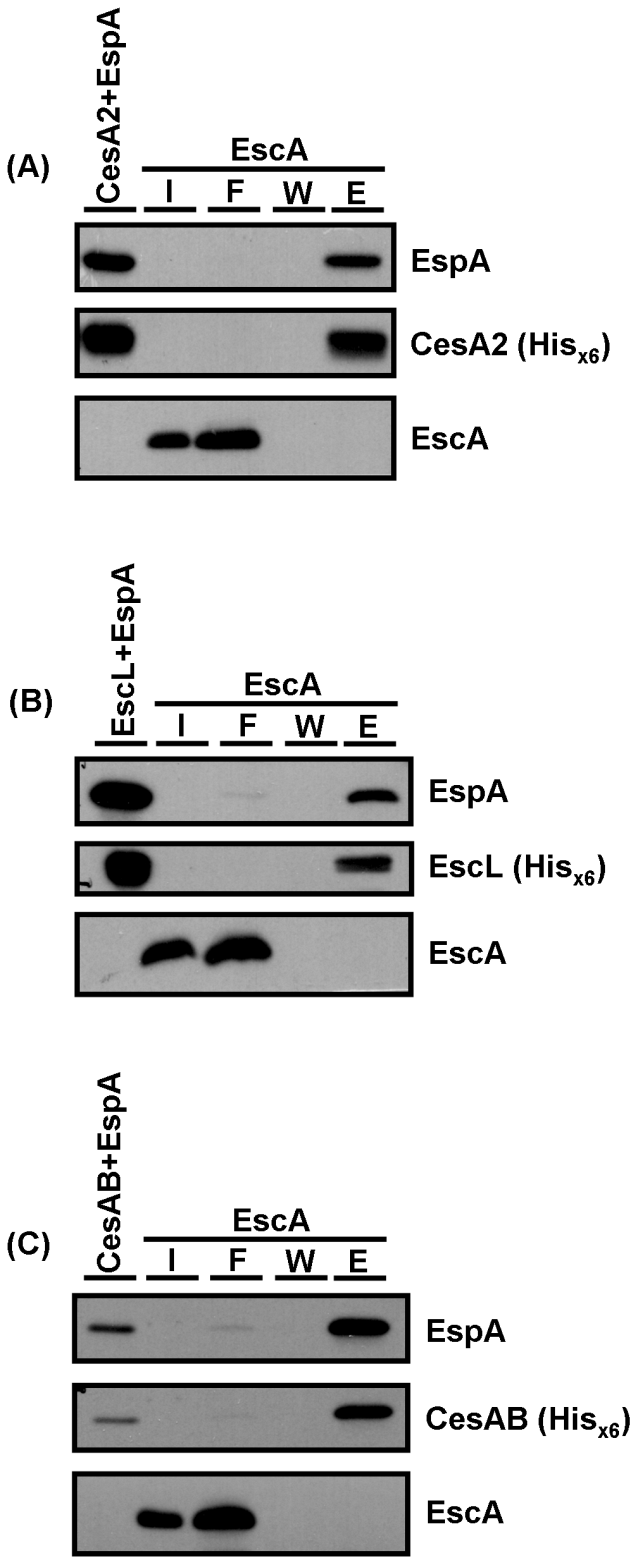
Neither disruption nor ternary multipart formation to the preformed complex of CesA2-EspA, EscL-EspA, or EscL-EspA by adding EscA. Experimental procedure was similar to Figure 4 except that tagged-free EscA was individually applied after the columns that were first bound with His_×6_-tagged proteins followed by binding of tag-free EspA. I: input; F: flow through; W: wash; E: eluent.

To unveil the second layer of possibility, we address whether stable interaction between EspA with CesA2 could be disrupted by the presence of EscA or a ternary complex could be formed after the addition of EscA. His_×6_-tagged CesA2 and tag-free EspA interaction was first performed in the Ni^2+^-NTA column. It was then followed by applying tag-free EscA in a clarified bacterial lysate into the column. After extensive washing, proteins co-retained with His_×6_-tagged CesA2 in the Ni^2+^-NTA column was examined by Western blotting analysis. Figure 5A shows clearly that EspA and CesA2 were detected in the eluent fraction but not EscA, a result suggesting that the complex of CesA2 and EspA remains stable and neither a ternary complex consisting of CesA2-EspA-EscA is stably formed after the addition of EscA. A similar experiment was performed by retaining His_×6_-tagged EscL and tag-free EspA first in the column followed by supplementing with EscA. Neither disturbing effect nor ternary multipart formation was seen with the addition of EscA (Figure 5B). A negative observation was also found when EscA was added into the column that first retained His_×6_-tagged CesAB and EspA (Figure 5C).

### Regulation of EscA expression

**Figure 6:**
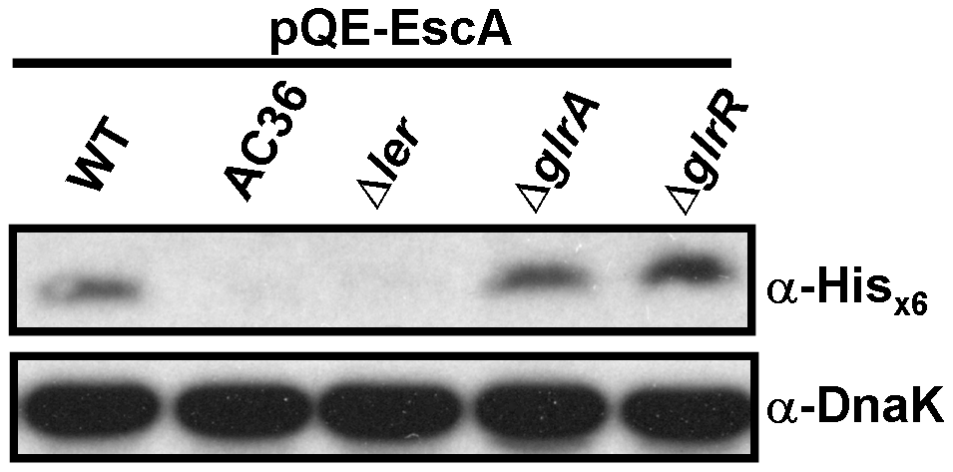
Lack of expression of EscA in the absence of Mpc (or Ler) in EHEC. Bacteria of different gene-deleted EHEC strains were transformed and induced to express EscA by IPTG. EscA from the total bacterial lysate was analyzed by Western blotting using rabbit anti-His_×6_. DnaK was used as a loading control.

To explore the possibility that there is an EscA regulatory mechanism in EHEC, we tested whether the expression of EscA was responding to the known gene deletions in LEE that grossly affect TTS of EHEC. We transformed pQE-EscA into individual EHEC variants that contain deletions affecting each of the known regulators in the LEE, namely *ler*, *glrA*, *glrR*, and *mpc*. We then examined the expression levels of EscA. Figure 6 shows that deleting *grlA* or *grlR* had no adverse effect on the expression level of EscA when compared to the parental WT strain. On the other hand, EscA could hardly be detected in the *ler* deletion strain (Δ*ler*). EscA was also absent in strain AC36, in which initiation of Mpc translation has been abolished, but transcription of the rest of *LEE3* remains untouched [18]. Since expression of EscA from pQE-EscA is governed by the exogenous promoter *T5*, it was reasoned that the above expression differences were unlikely to be due to transcriptional variation at the *escA* mRNA level between strain AC36 (or Δ*ler*) and strain Δ*grlA* or Δ*grlR*. Therefore, we proposed that EscA expression is correlated with the presence/absence of Mpc and/or Ler.

### Expressing regulation of EscA is mediated by interaction with Mpc

**Figure 7:**
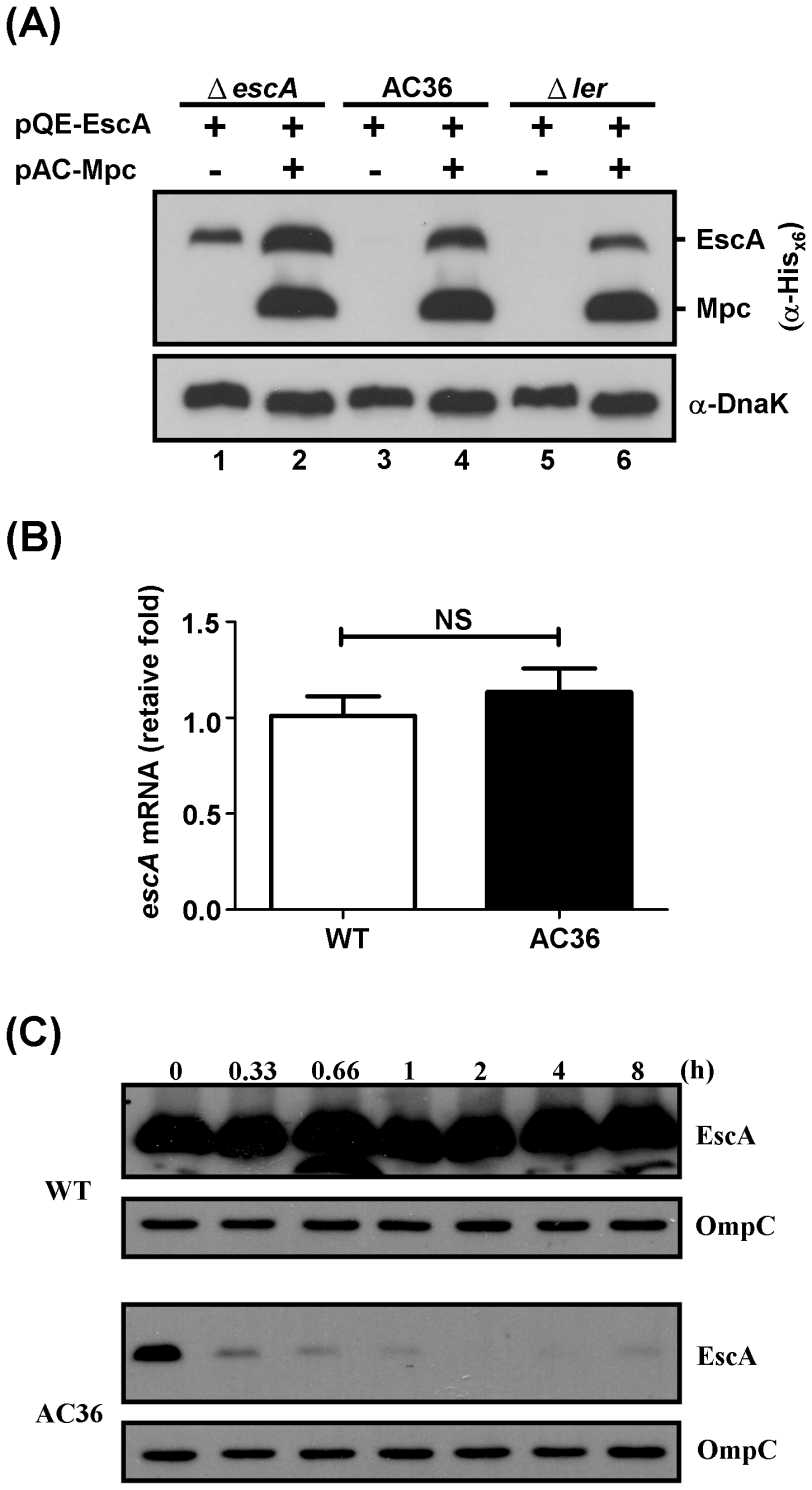
The presence of Mpc is critical for EscA expression. (A) Restoring the lost EscA-expression phenotype with strains AC36 and Δ*ler* by expressing Mpc ectopically. Compatible pAC-Mpc was co-transformed with pQE-EscA into bacteria and, then the bacterial lysates were examined for the expression of His_×6_-tagged EscA by Western blotting. Note: Mpc was also His_×6_-tagged and detected by the same rabbit anti-His_×6_ in Western blotting as EscA. (B) qRT-PCR analysis of *escA* mRNA expression level from WT and AC36 strains. Data were derived from triplicates. NS: non-significant at *p*<0.05. (C) Stability of EscA in the strains of WT and AC36 after bacteria receiving chloramphenicol. Experiments were carried out in a manner similar to that in Figure 2B. OmpC was used as a loading control.

To test the above possibilities, we examined the strains AC36 and Δ*ler* after supplementing with the critical element to determine whether correct EscA expression was recovered. Since Ler is a global LEE regulator that activates multiple *lee* operons when up-regulation is signaled, it would not be surprising that extra Ler was able to trigger an increase in expression of various LEE proteins including EscA. Therefore, we chose rather to ectopically express Mpc from a compatible plasmid pAC-Mpc. Mpc and EscA, both His_×6_ tagged, were co-expressed in strains AC36 and Δ*ler* (Figure 7A). As a reference, we included strain Δ*escA* in the experiments. As seen in Figure 7A, strain Δ*escA* that simultaneously harbored pQE-EscA and pAC-Mpc gave an increasing expression of EscA when compared to the same strain harboring pQE-EscA alone (compare lanes 1 and 2). Using strain AC36 (Figure 7A, lane 3) and as seen in Figure 6, no EscA was detected when this strain was transformed with pQE-EscA alone. However, when pAC-Mpc striking results were seen, however, when the Δ*ler* strain was transformed with pQE-EscA; this gave no detectable EscA as seen above (Figure 6). Co-transformation with both pQE-EscA and pAC-Mpc resulted in significant expression of EscA (Figure 7A, comparing lanes 1, 5, and 6). Since *mpc* is the first gene in the *LEE3* operon and *escA* is was co-transformed with pQE-EscA into this strain, this gave identifiable EscA expression (Figure 7A, lane 4). More located downstream of *mpc*, it seems likely that Mpc has a direct effect on the expression of EscA.

To examine whether the amounts of mRNA coding for EscA differs between strain AC36 and the WT strain, RNAs from both strains were extracted and analyzed by qRT-PCR. Results in Figure 7B indicated that the levels of *escA* mRNA had no significant difference between the WT and AC36 strains. The notion that RNA level difference could not account for the adverse dissimilarity between the expression levels of the two strains could be further supported by the experiments in Figure 7A, in which mRNA for EscA was driven all by the *T5* promoter. Therefore, regulations that may affect the protein *per se* were further examined. A careful examination of EscA detected in the pQE-EscA-transformed WT and AC36 strains was then carried out. Figure 7C shows the sequential detection results of EscA after that bacteria received chloramphenicol and previously induced with IPTG for 0.5 h. EscA level in the Mpc-deficient strain (AC36) was obviously lower than that in the WT strain at time zero, and the EscA signal disappeared quickly and was barely detected after 1-h bacterial culture. On the other hand, EscA protein in WT was relative stable and it remained abundant during the assay period (8 h). The above results suggest that the protein stability of EscA may account for the major difficulty to express EscA in the absence of Mpc. A simple hypothesis is that Mpc might interact with EscA and enhance EscA expression.

**Figure 8:**
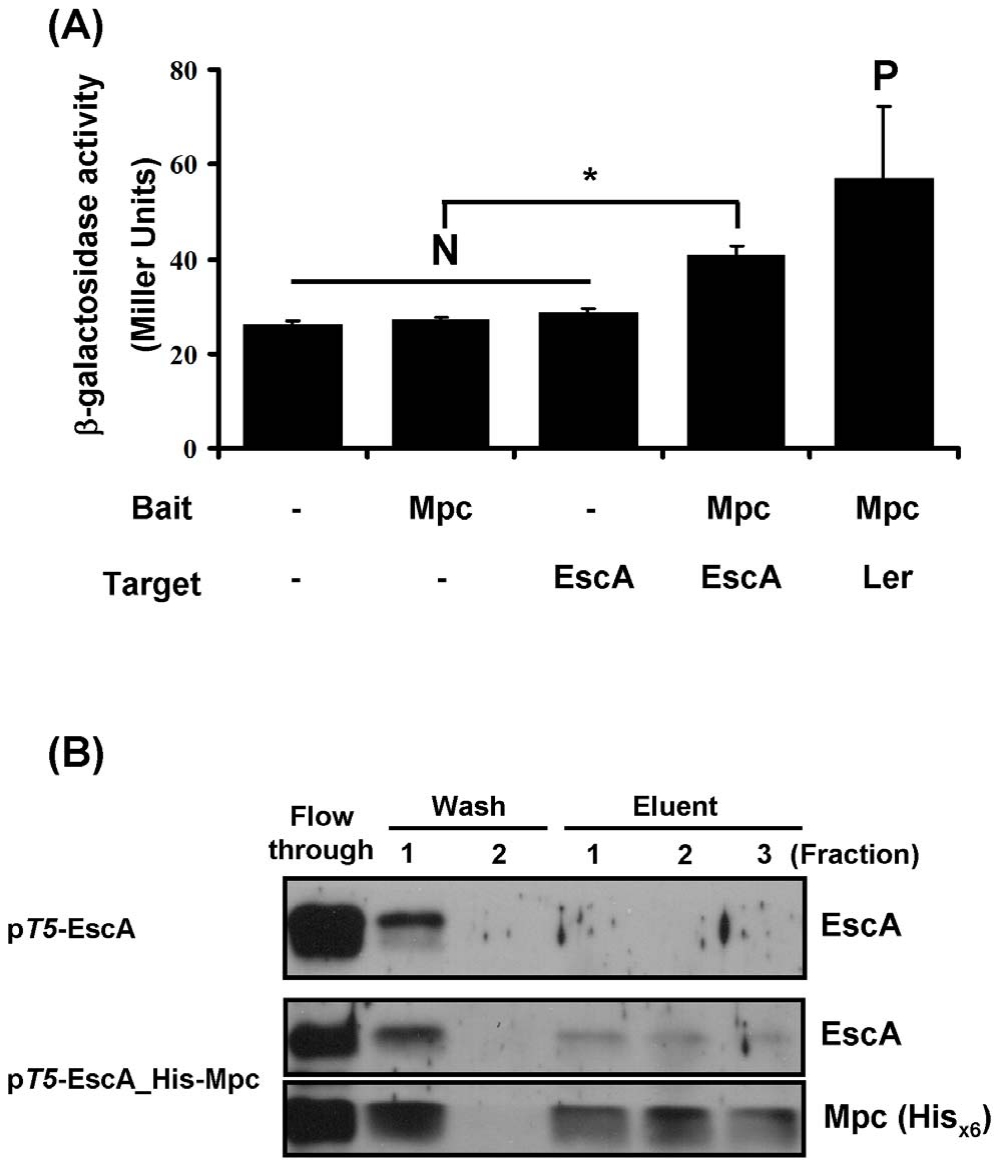
Mpc directly interacts with EscA. (A) Interaction between EscA and Mpc demonstrated by the bacterial two-hybrid system. Mpc was cloned as a bait fusion protein whereas EscA was cloned as a target [18]. Interaction of the bait and the target is reflected as an increase in β-galactosidase activity when compared to the negative (N) control. Interaction between Mpc and Ler was used as a positive (P) control. *: *p*<0.001 (B) Co-elution of EscA with Mpc. The experiments were carried out in a manner similar to that of Figure 3.

To test the above notion, we examined the possible interaction between EscA and Mpc using a bacterial two hybrid system. In this system, interaction between target protein and bait protein is revealed by way of an increase in reporter β-galactosidase activity. As references, three negative controls and one positive control [18] were included in the experiments. The results in Figure 8A showed that EscA does interact with Mpc, albeit not strongly. A reciprocal hybrid, where Mpc was incorporated as the target protein with EscA acting as the bait protein, also gave a positive result (data not shown).

To confirm the above notion that Mpc interacts with EscA, a co-elution assay was also performed. Using a strategy similar to that seen in Figure 3, we generated expression plasmids expressing tag-free EscA alone and with His_×6_-tagged Mpc simultaneously. After eluting His_×6_-tagged Mpc from the Ni^2+^-NTA column, the fractions were examined for the presence of EscA. Figure 8B confirms that EscA is readily co-eluted with Mpc but the protein was absent from the eluent when no His_×6_-tagged Mpc was present in the system.

## Discussion

EscA is a small protein rich in coiled-coil structures. Previous studies have shown that EscA is indispensable for the secretion of both translocator and effector in EPEC and *C. rodentium* [8], [9]. It has also been shown that EscA interacts with EscC [9], one of the TTSS components on the outer membrane that protrudes toward the periplasmic space. And EscA has been found mainly in the periplasmic fraction, followed by inner membrane-associated fraction and then in the cytoplasm. The property whereby EscA is found in is consistent with the notion that EscA makes a “contribution to the structural integrity of the TTS complex” [9] and backs the physical necessity that EscA interacts with periplasm-protruding EscC. As seen from our findings, EscA also interacts with a number of other LEE proteins in addition to EscC. First, EscA is able to interact independently with EscL and CesA2, two of the three currently known EspA-binding LEE proteins. It is worth noting that, EscL and CesA2 have been localized to the inner membrane-associated fraction [23], [26], a place where EscA is also detected. In contrast, CesAB is found in the bacterial cytoplasm and functions as an EspA chaperone in that compartment. However, this EspA-binding protein EscA is unable to interact with CesAB. The fact that EscA by itself is able to interact with EspA means that EscA is the fourth EspA-binding protein. Furthermore, it shares in common with the other EspA-binding proteins the fact that deletion of the gene coding for the cognate EspA-binding protein decreases the stability of EspA and this reduces the intracellular level of EspA. Therefore, EscA readily fits into this category.

**Figure 9:**
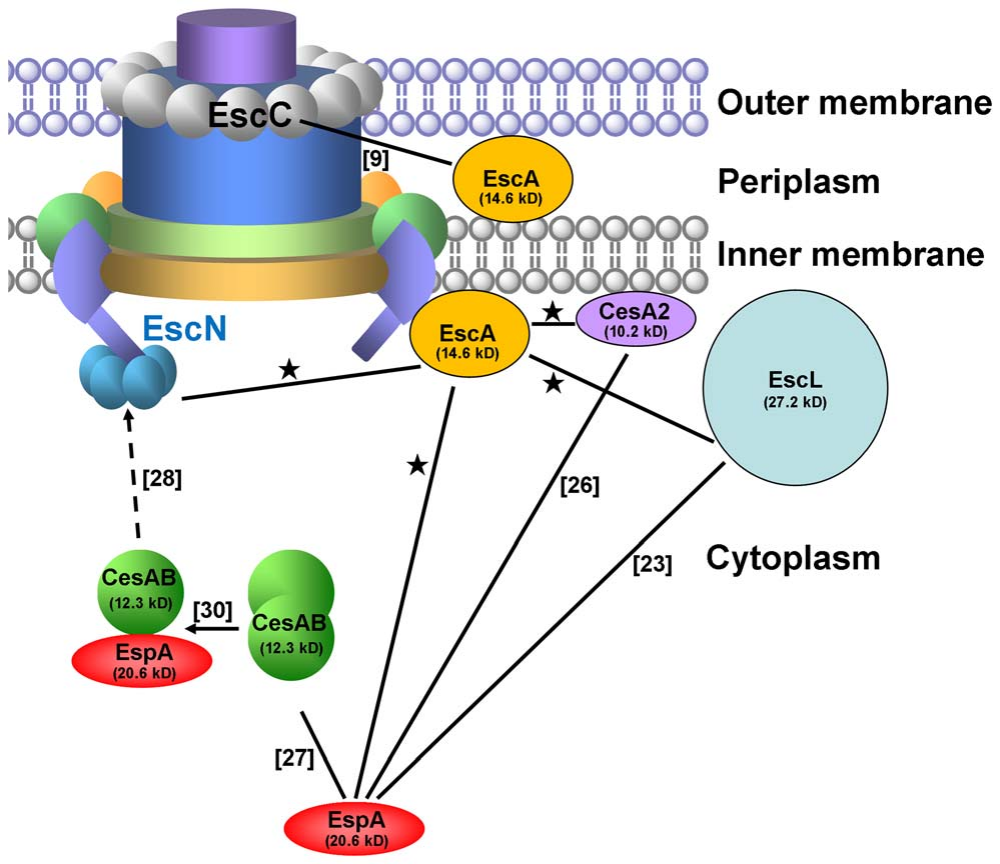
A summary of networked interactions of currently known EspA-binding proteins. Three of the four known EspA-binding proteins are associated with the inner membrane, as shown near a basal body of model TTSS [15]; they are EscL, CesA2, and EscA. EscA alone is able to interact with EscN ATPase, an event that characteristically differs from the interaction between CesAB and EscN, which requires a pre-exposure of CesAB to EspA [28]. EscA also interacts with EscC that is localized to the bacterial outer membrane, perhaps due to that EscA is also present in the periplasm [9]. Solid line indicates an interaction previously suggested, with reference provided; interaction suggested here is asterisked. Broken arrow depicts that a conformational switch of CesAB is needed before CesAB interacts with EscN and the switch is triggered by EspA (linked by an arrow line). Note: EscA does not disrupt the binary interactions of EspA with the other EspA-binding proteins nor it stimulates a ternary complex formation from the existing binary interaction complexes.

Before being exported out, one rationale is that secretory effector proteins should be quenched with chaperons to keep them in the unfolded status. Once the TTSS is activated, these proteins should be escorted by chaperons from the cytosol to cytoplasmic base of an injectisome, a membrane-associated basal apparatus. EscN, an essential ATPase for TTSS locating to the entrance of the injectisome, provides the energy to disassemble the chaperon/effector protein complex in an ATP-dependent manner [29]. Exemplified is that homodimeric CesAB does not interact with EscN. However, upon interaction with EspA, CesAB homodimer transforms into CesAB/EspA heterodimer that also induces a conformational switch of CesAB [28], [30] so that CesAB is able to interact with EscN by exposing the EscN-recognition domain. Here, we found that EscA alone is able to interact with EscN, an observation that is rather similar to the interaction between CesT and EscN [31]. To sum it up, currently known interactions among EspA and its binding proteins are depicted in Figure 9.

Amazingly seen is that EscA is able to interact with Mpc, a protein encoded by the first gene of the *LEE3* operon, in which *escA* is the fourth open reading frame (Figure 1A). Mpc has been previously shown to be indispensable but is expressed at only a low level since strong expression results in a severe suppression of the TTSS via counteraction with Ler [18]. Here, we have demonstrated that Mpc is essential for the expression of EscA (Figure 7). In terms of *LEE3* organization, *escA* comes after *mpc*, *escV*, and *escN* in the operon and before *l0032*, *sepQ*, and *espH*. The indispensable role of *mpc* in EscA expression and the interaction of Mpc with EscA suggest to us that EscA also needs to be expressed at a low level and EscA degrades rapidly without the presence of Mpc. It is then conceivable that such expression needs to occur in a timely manner for the purpose of successfully assembling the basal body of the TTSS. To test whether EscA is able to suppress the synthesis of TTSS proteins in a similar manner to Mpc, EscA was over-expressed in the WT strain of EHEC. The results (data not shown) indicate that an increased expression of EscA seems to have no apparent effect on the TTSS. Thus, it is likely that EscA does not play as profound a regulatory role in the LEE as Mpc.
